# Development and validation of a reverse transcription-insulated isothermal PCR assay for detection of caprine kobuvirus and its epidemiological and molecular characterization in China

**DOI:** 10.3389/fvets.2026.1915957

**Published:** 2026-10-05

**Authors:** Yang Su, Wangqing Banma, Guangfu Zhao, Qibing Gu, Falong Yang, Shenglin Li, Erzi Qubi, Kebu Munai, Musha Jili, Yutao Shi, Mingjing Gu, Yaoxuan Li, Junwen Wang, Kehamo Abi

**Affiliations:** 1 Key Laboratory of Veterinary Medicine of Universities in Sichuan, Southwest Minzu University, Chengdu, China; 2 Animal Disease Prevention and Control Center of Aba Prefecture, Barkam, China

**Keywords:** caprine kobuvirus, diarrhea, goats, iiPCR, molecular characteristics

## Abstract

Caprine kobuvirus (CKoV) is an emerging diarrheal pathogen in goats. We developed and validated a reverse transcription-insulated isothermal PCR (iiPCR) targeting the conserved 3D gene for detection of CKoV. The assay demonstrated high analytical specificity, a limit of detection of 39.9 copies·μL^−1^, and good repeatability. A total of 722 goat fecal samples were collected from five farms in Sichuan, Chongqing, Yunnan, and Hunan in China and tested using the newly developed method. The overall CKoV positivity rate was 40.44% (292/722), and the farm-level positivity rate was 100% (5/5), with the highest rate in Hunan (84.00%, 21/25), followed by Yunnan (42.86%, 48/112), Sichuan (38.58%, 206/534), and Chongqing (33.33%, 17/51). Lambs exhibited substantially higher positivity rates (57.14–83.33%) than goats aged ≥3 months (13.39–15.52%), and chi-square test confirmed a statistically significant association between age and CKoV positivity (*χ*^2^ = 252.97, *df* = 6, *p* < 0.001), confirming age-dependent susceptibility in lambs. Twelve full-length VP0 and VP1 sequences were obtained (GenBank: PZ424409-PZ424432). Phylogenetic analysis revealed that all isolates formed an independent clade related to known CKoV strains. The VP0 sequences shared 97.28–100% amino acid identity, while the VP1 sequences shared 98.61–100% identity. Compared with the reference strain (GenBank: MT584793.1), stable substitutions were found in the VP0 receptor-binding domain and a conserved VP1 region, potentially affecting protein conformation or function. This study provides a validated detection tool and new insights into CKoV epidemiology, genetic diversity, and evolution.

## Introduction

Kobuviruses (family Picornaviridae) are important enteric pathogens associated with acute gastroenteritis in humans and various animals (1–7). This genus currently comprises six officially recognized species, Aichivirus A-Aichivirus F classified on the basis of host range and pairwise genomic homology (5–7). Since the prototype Aichivirus A (formerly Aichi virus) was first identified in patients with acute gastroenteritis in Japan in 1989 (1), kobuviruses have subsequently been detected in a wide range of hosts, including pigs, sheep, goats, cattle, and other species (2–5). Caprine kobuvirus (CKoV) was first reported in diarrhoeic black goats in South Korea in 2012 (4, 5) and has since been detected in clinically healthy goats in Italy, USA and China (8–10). Furthermore, genetically related strains recovered from free-ranging roe deer (*Capreolus capreolus*) in Italy suggest that CKoV may be capable of crossing the species barrier, underscoring the need for surveillance of interspecies transmission (11).

Several molecular detection methods for CKoV have been reported (5, 8–10). Among these, two assays employ universal primers targeting the conserved 3D gene, relying on PCR and sequencing for species identification; however, their specificity and efficiency require further validation (8, 10). The specific RT-PCR assay described for CKoV is prone to primer-template mismatches when applied to Chinese strains (e.g., nucleotide substitutions within the primer-binding regions), likely compromising its amplification efficiency (5). To date, no real-time fluorescent quantitative PCR (qPCR) method has been reported for CKoV. Furthermore, none of the existing assays are suitable for point-of-care testing due to their lack of portability.

Insulated isothermal PCR (iiPCR) is a rapid, simple nucleic acid amplification technique widely used for point-of-need detection of various veterinary and zoonotic pathogens (12–20). In this study, we developed and validated a TaqMan-based real-time iiPCR assay targeting the conserved 3D gene of CKoV, enabling field-deployable detection of this pathogen. Using this assay, we screened 722 diarrheal samples collected from five farms to determine the prevalence of CKoV in China. To investigate the genetic diversity of circulating strains, we amplified and sequenced the full-length VP0 and VP1 genes from 12 CKoV-positive samples. Our findings provide a robust molecular tool for CKoV surveillance and offer new insights into the genetic diversity and evolutionary characteristics of this emerging pathogen.

## Materials and methods

### Primer and probe design and synthesis

The primers and probe were designed using Premier 5.0 software based on the 3D gene sequences of CKoV. To ensure broad sequence coverage, we downloaded all 24 available CKoV 3D gene sequences from GenBank (19 partial and 5 complete sequences) and compared their sequences. The sequence information is provided in Supplementary material. Based on this comparison, a 136 bp segment (GenBank accession number MW187495.1) was selected as the target region because it exhibited the highest sequence homology among the analyzed sequences. The new primer sequences were as follows: F: 5′-CAAACTAACTGGAGATGAAA-3′, R: 5′-AGTGTTGATGATGGAAGTA-3′; and the TaqMan probe sequence was 5′-FAM-TGCCGCCAATCATCTCATACG-BHQ1-3′, with FAM at the 5′ end and BHQ1 at the 3′ end… Shanghai Sangon Biotech Co., Ltd. was responsible for synthesizing the primers and probes.

### Nucleic acid extraction

Fecal samples were suspended in phosphate-buffered saline (PBS) at a 1:5 ratio and centrifuged at 5,000 ×*g* for 8 min. The supernatant was filtered through a 0.22 μm filter to remove debris. RNA from CKoV, other RNA viruses, and clinical samples was extracted using Trizol; DNA from DNA viruses and bacteria was extracted using phenol-chloroform. The isolated RNA and DNA were stored at −80 °C and −20 °C, respectively. Then, reverse transcription was performed using the All-in-One 5X RT MasterMix (ABM, Canada) according to the manufacturer’s instructions. The reaction program was set as follows: 37 °C for 15 min, followed by 60 °C for 10 min, and a final inactivation step at 95 °C for 3 min. The resulting cDNA was used as a template for the subsequent iiPCR assay.

### Preparation of CKoV-positive reference material

A CKoV standard plasmid was prepared, and its copy number was quantified. The 136 bp 3D-pol fragment amplified from CKoV-cDNA was cloned into the pMD19-T vector and sequenced. The copy number was calculated using the formula: copies/μL = [plasmid concentration (ng/μL) × 10^−9^ × 6.022 × 10^23^]/[660 × plasmid size (bp)].

### Optimization of the reaction system

To optimize the iiPCR assay, the CKoV positive standard plasmid (61.5 ng/μL, 3.99 × 10^10^ copies/μL) was used as template and positive control, with DEPC-treated H₂O as negative control. At the optimal template volume of 1.2 μL, the positive control contained approximately 4.79 × 10^10^ copies per reaction. The iiPCR assay was performed on a POCKIT Micro Nucleic Acid Analyzer (GeneReach Biotechnology Corp., Taichung, Taiwan), and the results were automatically displayed as positive (+) or negative (−). The optimal conditions were defined as the minimum reagent volumes that consistently produced a positive result for the positive control and a negative result for the negative control.

### Analytical specificity evaluation

To determine the analytical specificity of the established iiPCR method, samples positive for Torovirus, Enterovirus, Bopivirus, Bocaparvovirus, Astrovirus, *Escherichia coli*, and *Salmonella* were tested as additional targets. The prepared CKoV positive standard plasmid was used as a positive control to confirm the correct functioning of the reaction system, whereas DEPC-treated H_2_O was used as a negative control to monitor for contamination. The absence of amplification signals in the additional targets confirmed the high specificity of the iiPCR assay.

### Analytical sensitivity test

To determine the detection limit of this method, the CKoV positive standard plasmid (61.5 ng/μL, 3.99 × 10^10^ copies/μL) was serially diluted 10-fold from 10^−1^ to 10^−11^ (3.99 × 10^9^ to 0.399 copies/μL), and each dilution was tested by the established iiPCR method.

### Repeatability evaluation

To assess the intra-assay repeatability of the established iiPCR method, the CKoV positive standard plasmid (61.5 ng/μL, 3.99 × 10^10^ copies/μL) was serially diluted 10-fold, and seven dilutions (1 × 10^−2^ to 1 × 10^−8^, corresponding to 3.99 × 10^8^ to 3.99 × 10^2^ copies/μL) were tested in triplicate within a single run. DEPC-treated H_2_O was used as the negative control.

### Comparison of the five methods

A convenience panel of 28 fecal samples was collected from lambs in a sheep farm in Mianyang, Sichuan, China, on April 15, 2025, during an outbreak of severe diarrhea. The lambs selected for this panel showed severe diarrhea and marked depression/dullness. Fecal samples were transported to the laboratory and processed immediately upon arrival. No CKoV-specific laboratory pre-screening was performed before sample selection. These samples were used only for comparison of the iiPCR assay established in this study with four previously reported RT-PCR methods (5, 8–10) and were not randomly selected. The detailed primer sequences, reaction mixtures, amplification conditions, and PCR mechanisms for the four conventional RT-PCR methods are provided in Supplementary material.

### Detection of CKoV in clinical samples

The iiPCR method established in this study was used to screen a total of 722 clinical fecal samples collected from goats with diarrhea from April 2025 to March 2026 from four farms (two farms in Sichuan, *n* = 534; one farm in Chongqing, *n* = 51; one farm in Yunnan, *n* = 112; one farm in Hunan, *n* = 25) for the presence of CKoV.

### Amplification and analysis of the complete VP0 and VP1 gene sequences of CKoV

Two pairs of primers were designed based on the target gene sequences of KoV. The primers used for amplifying the complete VP0 and VP1 genes from clinical fecal samples are as follows: Primer 1 (F: 5′-GAATGTCCCACTTGTGACCCC-3′, R: 5′-CACAGGGATTTCTTGTCCAGC-3′) amplified a 1,167 bp fragment covering the complete VP0 gene; Primer 2 (F: 5′-CTTTGTTTCTGCTGGGGAG-3′, R: 5′-AATTTGTTGCACACCGCTC-3′) amplified a 942 bp fragment covering the complete VP1 gene. All primers and amplification products were synthesized and sequenced by Shanghai Sangon Biotech Co., Ltd. The resulting sequences were assembled using SeqMan software, and sequence alignments were compared with GenBank sequences via BLAST alignment. A phylogenetic tree was constructed using MEGA 12.0 software with the Neighbor-joining (NJ) method and bootstrap values calculated for 1,000 replicates.

## Results

### Establishment and evaluation of iiPCR for detection of CKoV

#### Optimization of reaction conditions

The optimal amplification conditions for the iiPCR assay were established using a 50 μL reaction volume consisting of 25 μL of Premix Ex Taq™, 3.5 μL of each primer (10 μmol/μL), 2 μL of probe (10 μmol/μL), 2 μL of template, and an appropriate volume of ddH_2_O.

#### Analytical sensitivity

Using a positive control with a mass concentration of 61.5 ng/μL and a copy number of 3.99 × 10^10^ copies/μL, the established iiPCR assay detected the target at 10^−9^ (39.9 copies/μL) but not at 10^−10^ (3.99 copies/μL) and 10^−11^ (0.399 copies/μL). Therefore, the minimum detection limit was defined as 39.9 copies/μL (10^−9^ dilution).

#### Analytical specificity

The iiPCR assay specifically amplified the target CKoV sequence. Positive amplification signals were only observed in CKoV-positive samples, while no amplification signal was detected in samples containing Torovirus, Enterovirus, Bocapivirus, Bocaparvovirus, Astrovirus, *Escherichia coli*, *Salmonella*, or nuclease-free water.

#### Repeatability

To evaluate repeatability, seven dilutions of the positive standard ranging from 1 × 10^−2^ to 1 × 10^−8^ were each tested three times, and the results were consistent, indicating the method has good repeatability.

### Comparison of five detection methods

A total of 28 clinical diarrheal fecal samples from goats were simultaneously tested for CKoV using the iiPCR method established in this study and four other reported detection methods. The detection rate of the iiPCR method was 96.43%, higher than the other four methods ranging from 0 to 89.29%. Notably, all positive samples identified by the conventional methods were also detected by iiPCR, and sequencing confirmed all amplified products matched the target CKoV gene fragment. These results indicate the newly established iiPCR possesses superior applicability for rapid clinical screening of CKoV.

### Detection of CKoV in clinical samples

Out of the 722 fecal samples, 292 (40.44%) were identified as CKoV positive by the iiPCR method established in this study. Among them, the positive rate was the highest in Hunan Province, reaching 84.00% (21/25), followed by Yunnan Province at 42.86% (48/112), Sichuan Province at 38.58% (206/534) and lastly Chongqing Municipality at 33.33% (17/51). The sample information is shown in the Figure 1. Notably, lambs had higher rates: 83.33% (55/66) at 3 days, 80.30% (53/66) at 5 days, 78.46% (51/65) at 7 days, 57.14% (36/63) at 10 days and 62.50% (40/64) at 1 month, while the positivity rate in goats aged 3 months and older was only 15.52% (27/174) and 13.39% (30/224) (Table 1). To evaluate the association between age groups and CKoV positivity, a Chi-square test was performed, which revealed a highly significant difference among the age groups (*χ*^2^ = 252.97, *df* = 6, *p* < 0.001). Post-hoc pairwise comparisons with Bonferroni correction for multiple testing (adjusted α = 0.05/21 = 0.00238) showed that lambs aged 3–10 days and 1 month had significantly higher positivity rates than goats aged ≥ 3 months (*p* < 0.001), while no significant difference was observed between the 3-month-old and ≥3-month-old groups. The positivity rates exhibited a clear age-dependent pattern, with the highest rates observed in 3-7-day-old lambs (78.46–83.33%), a gradual decline at 10 days of age (57.14%), and a slight increase at 1 month of age (62.50%), which may be associated with the weaning period and waning maternal antibodies. These findings indicate that there is epidemiological significance of CKoV, demonstrating its widespread and severe prevalence across the surveyed regions.

**Figure 1:**
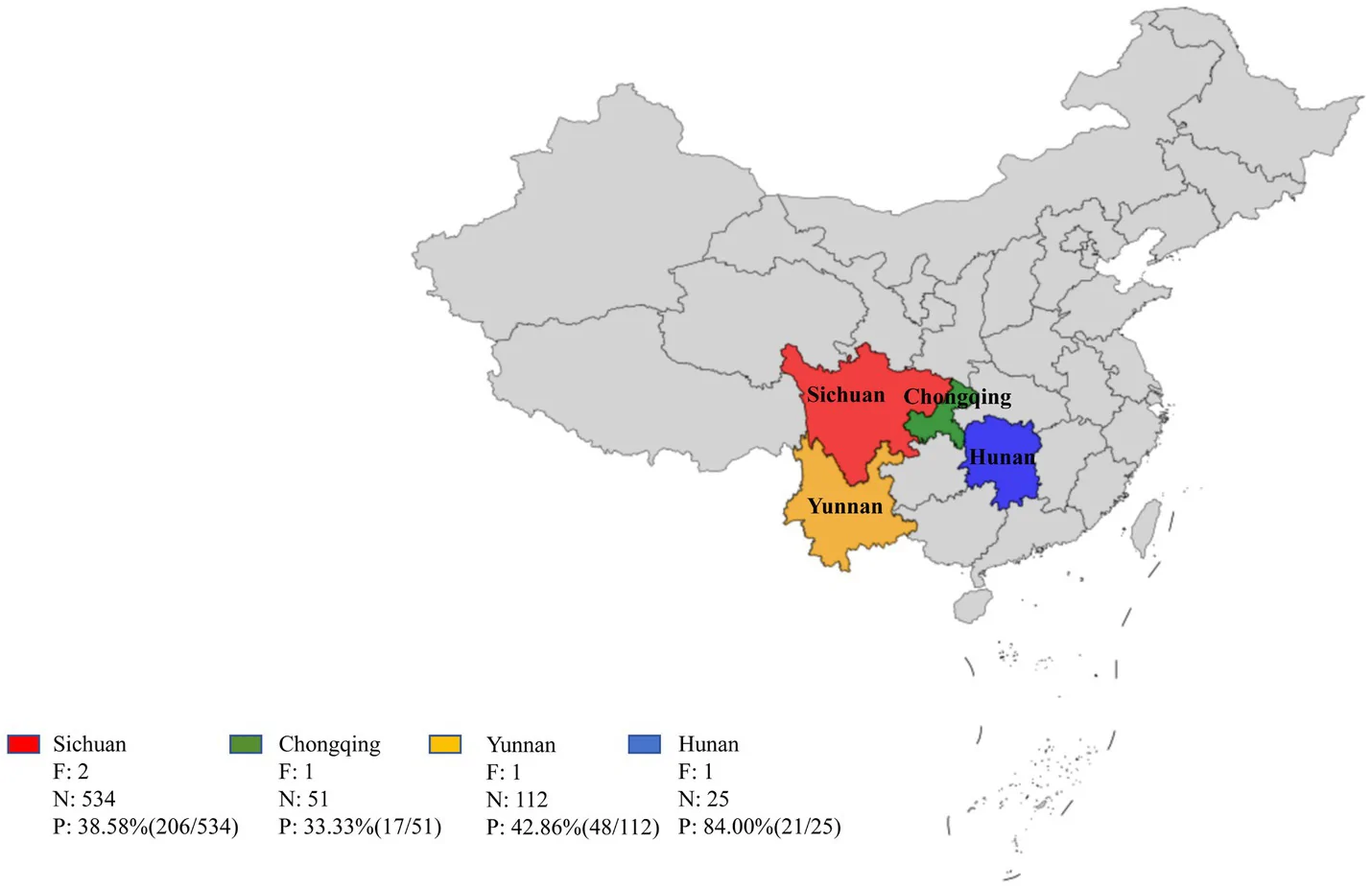
Number and positivity rate of samples from four regions in China, 2025–2026. F represents the number of goat farms, N represents the number of fecal samples, and P represents the positivity rate of CKoV.

**Table 1:** CKoV test results.

Sample type	Sample size	Positive rate(%)	95% CI	χ^2^ contribution
3 Days old	66	83.33% (55/66)	73.5–93.2	41.16
5 Days old	66	80.30% (53/66)	70.8–89.8	35.91
7 Days old	65	78.46% (51/65)	68.4–88.5	31.95
10 Days old	63	57.14% (36/63)	44.9–69.4	2.49
1 Month old	64	62.50% (40/64)	50.6–74.4	6.56
3 Months old	174	15.52% (27/174)	10.1–20.9	2.31
3 Months or older	224	13.39% (30/224)	8.9–17.9	16.35
Total	722	40.44% (292/722)	36.8–44.1	

### Molecular characteristics of the VP0 and VP1 genes

In this study, 12 full-length VP0 and VP1 sequences were obtained and deposited in GenBank under accession numbers PZ424409-PZ424432. Phylogenetic analysis revealed that all 12 isolates formed an independent clade closely related to known CKoV strains (Figure 2). The 12 VP0 sequences shared 97.28–99.64% nt identity and 97.28–100% aa identity among themselves, and compared with the reference strain CKoV-China/SWUN/F11/2019 (GenBank: MT584793.1), all amplified sequences exhibited 93.01–93.91% nt and 96.73–98.37% aa identity, and harbored five stable amino acid substitutions (A60S, S149P, I252L, T267V, I331V). Notably, I252L is located within the receptor-binding domain (RBD, 200–261 aa) of VP0 and may influence virus-receptor interactions, although functional validation is required. The 12 VP1 sequences showed 98.50–100% nt identity and 98.61–100% aa identity among themselves, and compared with the reference strain CKoV-China/SWUN/F11/2019, all amplified sequences shared 89.12–89.70% nt and 96.53–96.88% aa identity, and harbored eight stable amino acid substitutions (N57S, T244V, V248A, A251S, H252R, L255M, S257T, F283Y). Notably, six of these substitutions were clustered within the 244–257 region, suggesting a conserved mutation hotspot that may exert synergistic effects on protein conformation or function.

**Figure 2:**
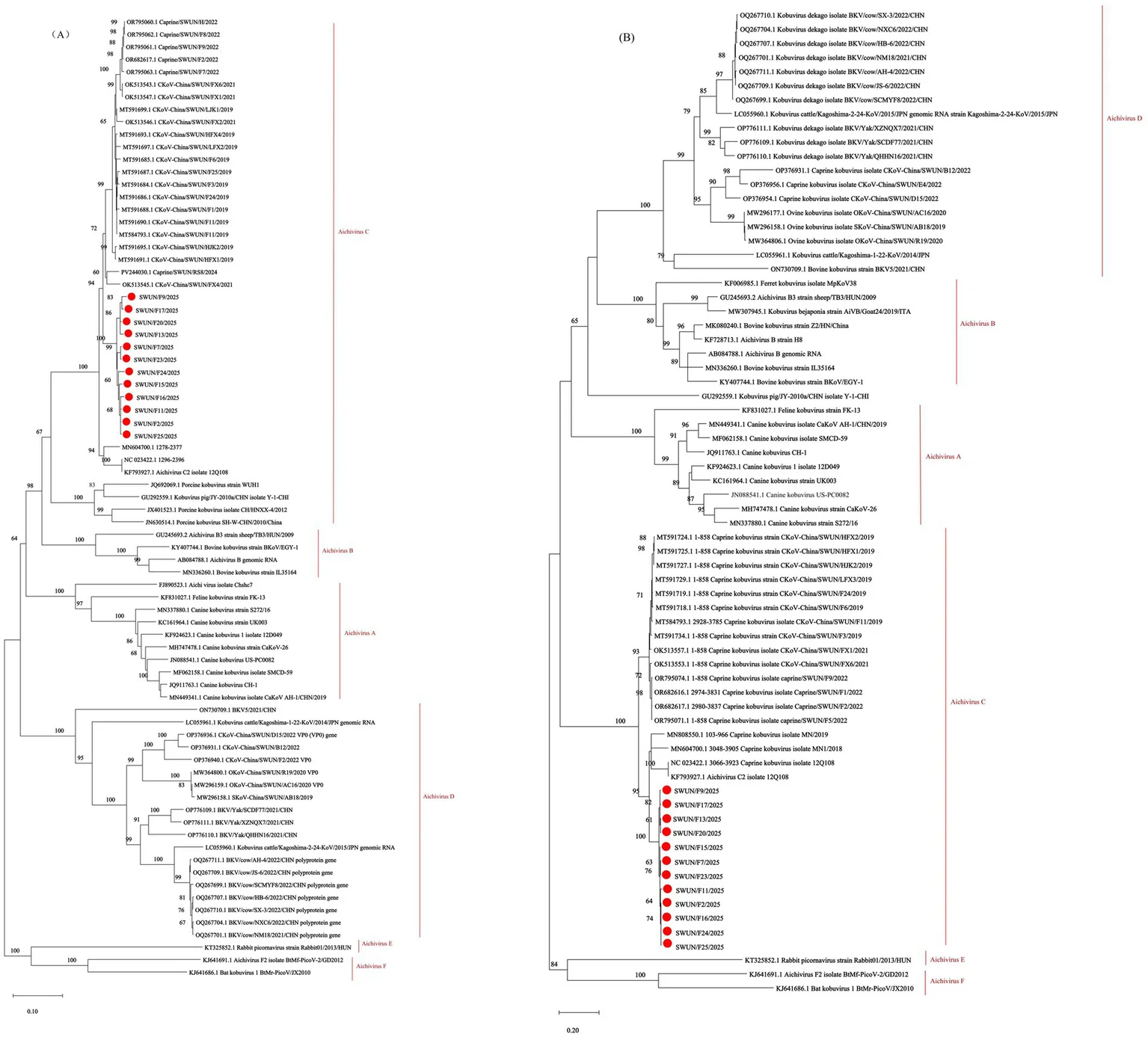
Phylogenetic relationships among CKoV and representative species of the genus Kobuvirus, based on the amino acid sequences of the complete VP0 **(A)** and VP1 **(B)** regions. Sequence alignment was performed using ClustalW, and the phylogenetic tree was constructed via the neighbor-joining (NJ) method in MEGA 12.0 with 1,000 bootstrap replicates. ● Denotes strains amplified in this study.

In this study, we compared the 12 amplified complete VP1 sequences with those of human Aichivirus in the antigenic peptide and receptor recognition domain regions. The results showed (Figure 3) that all sequences exhibited amino acid differences at the same positions in the antigenic peptide region, though the specific substitutions were not identical. In the receptor recognition domain, all sequences uniformly carried PRPPPPPLGAPPSA, whereas the human counterpart displayed PRPPPPPLPPLPTP, a polyproline motif known to be involved in receptor binding in human Aichi virus. These amino acid substitutions suggest a potential impact on virus-receptor interaction, although whether receptor binding is indeed affected requires further experimental validation.

**Figure 3:**
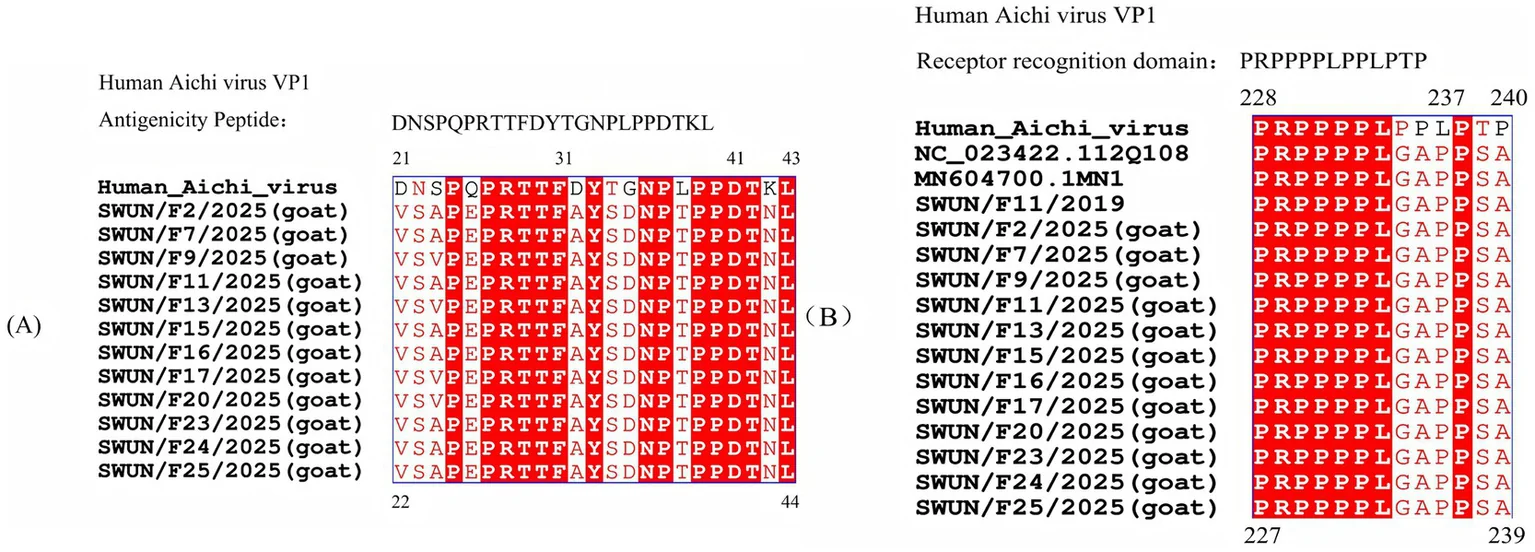
Amino acid variations in the antigenic peptides **(A)** and receptor recognition domains **(B)** of 12 complete VP1 genes.

The phylogenetic tree was constructed based on 18 CKoV VP1 sequences from the GenBank database. The results showed that the 12 VP1 sequences obtained in this study formed an independent small evolutionary cluster clearly divided into two branches, indicating that the strains belonged to two different genotypes (Figure 4). This genotype diversity suggests the co-circulation of multiple lineages among local caprine populations, warranting further surveillance and functional studies.

**Figure 4:**
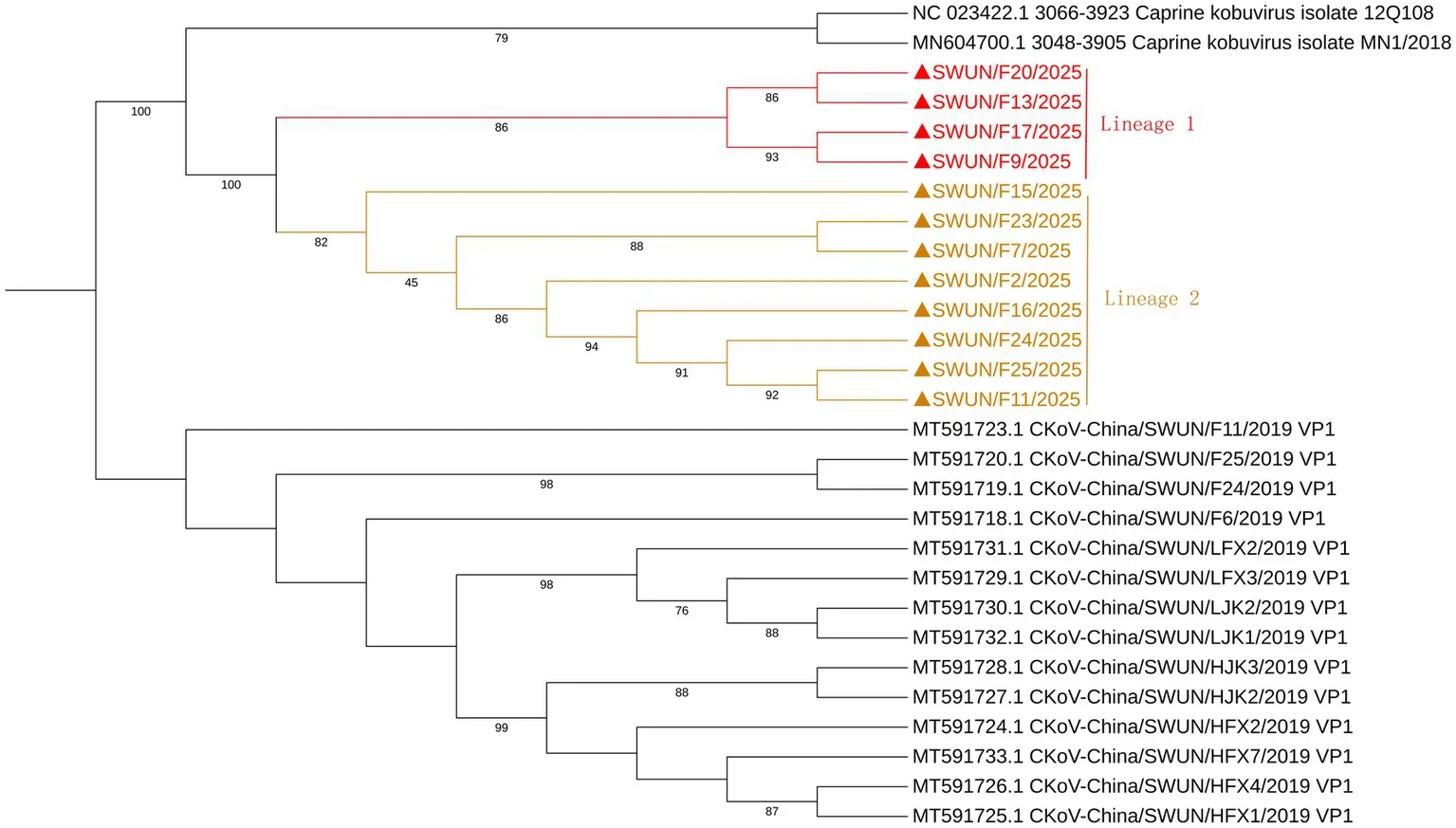
Phylogenetic tree of CKoV based on the VP1 sequences. Sequence alignment was performed using ClustalW, and the tree was constructed via the neighbor-joining (NJ) method in MEGA 12.0 with 1,000 bootstrap replicates. 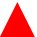 and 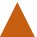 marks the strains identified in the present study.

## Discussion

In this study, a novel iiPCR assay for CKoV detection was successfully established and evaluated. The assay exhibited a detection limit of 39.9 copies·μL^−1^ and high specificity, with no cross-reaction against other common enteric pathogens. In comparison with four previously reported detection methods using 28 clinical diarrheal samples, the iiPCR assay demonstrated the highest detection rate (5, 8–10), and sequencing confirmed that all amplified products matched the target CKoV gene. These results indicate that the iiPCR method offers superior sensitivity and reliability. Given its isothermal amplification format, which eliminates the need for complex thermal cyclers, this assay represents a practical and cost-effective tool for rapid clinical screening and field surveillance of CKoV, particularly in resource-limited settings. Using the iiPCR assay on 722 fecal samples, we found an overall CKoV positivity rate of 40.44%, with marked regional variation. Notably, the prevalence observed in Sichuan Province in this study (38.58%) was considerably lower than the 87.5% (21/24) previously reported from the same region (9), while the overall positivity rate was significantly higher than the 5.8 and 2.6% rates reported in Italian goat populations (8, 21). These geographical disparities may be attributed to differences in sampling periods, herd management practices, and the sensitivity of detection methods. A strong age-dependent pattern was observed: lambs up to 1 month of age exhibited significantly higher infection rates compared to goats aged 3 months or older, consistent with kobuvirus infections in other livestock, where younger animals show greater susceptibility due to immune immaturity and waning maternal antibodies (22, 23). The relatively high overall prevalence of CKoV in this study suggests that CKoV is highly endemic in Chinese goat herds, which may be associated with intensive farming systems, high stocking density, and co-infections with other enteric pathogens. These findings underscore the importance of implementing enhanced surveillance programs and biosecurity measures, particularly in high-density farming areas and in lambs, to reduce the transmission and economic impact of CKoV infection. Phylogenetic analysis of 12 VP1 sequences revealed that all isolates formed an independent clade divided into two sub-branches, indicating two distinct genotypes co-circulating in the surveyed regions. The genotype classification shown in this study is based on the phylogenetic analysis of the complete VP1 sequences. Given that the complete VP1 gene is the standard target for genotype determination in kobuviruses, the distinct clustering pattern (two sub-branches) in the NJ tree indicates the presence of two distinct genotypes co-circulating within the CKoV species. This genetic diversity is consistent with the heterogeneity observed among caprine kobuvirus strains from Korea, Italy, and China (5, 8, 9). Notably, a novel Aichivirus D has also been recently identified in goats with diarrhea in China (24), further supporting the genetic diversity of kobuviruses infecting caprine populations. VP0 comparison identified five conserved substitutions, with I252L located within the receptor-binding domain (200–261 aa). Notably, this region corresponds to the VP0 CD loop (residues 195–253) of human Aichi virus, which has been proposed to play a role in receptor binding (25). VP1 analysis revealed eight substitutions, six clustered within residues 244–257, suggesting a conserved mutation hotspot that may affect protein conformation. Additionally, all caprine kobuvirus isolates carried the motif PRPPPPPLGAPPSA in the receptor recognition domain, while human Aichivirus possesses PRPPPPPLPPLPTP, a polyproline motif involved in receptor binding (25, 26). Comparison with the conserved VP1 epitope of Aichivirus (27) further supports the antigenic divergence between caprine and human kobuviruses, suggesting potential host-specific adaptation.

In conclusion, the iiPCR assay established in this study is a sensitive, specific, and reproducible method well-suited for clinical detection and epidemiological surveillance of CKoV. The epidemiological data reveal its widespread prevalence and a clear age-related susceptibility pattern, while the genetic data demonstrate significant heterogeneity and conserved mutation hotspots that may be relevant to viral evolution and host adaptation. The establishment of this rapid field-deployable assay provides a practical tool for timely diagnosis and surveillance, and the molecular and epidemiological findings offer insights into viral evolution, transmission, and host susceptibility, collectively laying a foundation for future studies on pathogenesis and vaccine development.

## Data Availability

The original contributions presented in the manuscript are included in the article/Supplementary material, further inquiries can be directed to the corresponding author.
