# Supplementary material for: Development and validation of a reverse transcription-insulated isothermal PCR assay for detection of caprine kobuvirus and its epidemiological and molecular characterization in China
Source: Front Vet Sci. 2026 Oct 5;13:1915957. doi: 10.3389/fvets.2026.1915957 (PMC13679536; doi:10.3389/fvets.2026.1915957)
Supplement: Supplementary file 1 [file Table_1.DOCX]

Supplementary Table SX (added to the Supplementary Materials):

1. Oem et al. [5]

Target gene: 3D

Primers: F: 5′-CATGCTCCTCGGTGGTCTCA-3′; R: 5′-GTCCGGGTCCATCACAGGGT-3′

Reaction mixture (25 μL): 3 μL template RNA, 15 μL 2× Reaction Mix, 0.6 μL each primer (10 μM), 1.2 μL SuperScript III RT, water to 25 μL

Thermal cycling: 55°C for 30 min; 94°C for 2 min; 40 cycles of 94°C for 30 s, 52°C for 30 s, 68°C for 1 min; 68°C for 5 min

PCR mechanism: RT-PCR targeting the conserved 3D gene of CKoV

2. Melegari et al. [8]

Target gene: 3D

Primers: F: 5′-GGATTACAAGTGTTTTGATGC-3′; R: 5′-ATGATGGTGTTGATGATCGAGG-3′

Reaction mixture and thermal cycling: same as Abi et al. [8]

PCR mechanism: RT-PCR targeting the conserved 3D gene of CKoV

3. Abi et al. [9]

Target gene: 3D

Primers: F: 5′-CGCCAAACCTGGAAGGCATCG-3′; R: 5′-GGTGGAGTAAAAGTAATCTGGGTTGGA-3′

Reaction mixture (20 μL): 10 μL 2×Universal Green PCR Master Mix (Rong Wei Gene, Cat. No. A303-1), 1 μL each primer (10 μM), 2 μL cDNA, 6 μL ddH₂O

Thermal cycling: 95°C for 3 min; 35 cycles of 95°C for 15 s, 58.7°C for 15 s, 72°C for 10 s; 72°C for 5 min; hold at 4°C

PCR mechanism: RT-PCR targeting the conserved 3D gene of CKoV

4. Sobhy et al. [11]

Target gene: 3D

Primers: F: 5′-TCAACTCTCTCTCAAACACACA-3′; R: 5′-GCCGCCAATCATCTCATAC-3′

Reaction mixture (25 μL): OneStep RT-PCR Kit (Qiagen)

Thermal cycling: 50°C for 30 min; 95°C for 15 min; 35 cycles of 94°C for 1 min, 50°C for 1 min, 72°C for 1 min; 72°C for 10 min

PCR mechanism: OneStep RT-PCR targeting the 3D gene of CKoV

Note: For Melegari et al. [8], the original publication did not provide complete reaction mixture and thermal cycling conditions. Therefore, the same conditions as described for Oem et al. [5] were used in this study.

1. Caprine kobuvirus isolate Caprine/SWUN/RS8/2024

ATCATCATTCCGGGACCATCCGTGGATCCCGGGGTGAATGTCAATCGCAAGTCCAAACTCGGTCGTTCCCCAGCTTTTGGTGCCTTTCCTGTCAAGAAACAGCCAGCCCCCCTCTCACAGAAGGACGAACGTCTCGAGGACGGGATCACCTTGGATGACCAACTCTTCCTTAAACACAACAAGGGAGATATGGATCAGCCATGGCCCGGTCTCGAGGCGGCCGCCGATCTTTACTTTTCTAAATTCCCCACTGTCATTCGAACCCTCACTCTCGAGGAGGCAATCAATGGGACGCCAAACCTGGAAGGCATTGACATGAATCAGGCTGCCGGGTATCCATGGAACACCATGGGACGCTCGCGCCGTTCTCTCTTTGTCGAACAGGCCCCTGGCTTCTACATGCCGACTCCGGAGTTGCAGGAGGAGATTGACAAAACCCTGTCCAACCCAGATTACTTTTATTCCACTTTTCTTAAAGACGAGCTCCGCCCGATCTCCAAAGTGACGGCGGGACTCACCAGAGTGGTCGAGGCTGCTCCTATACACGCTATCGTGGCCGGCCGCATGCTGCTCGGTGGACTCATCGAGTACATGCAGGCACATCCTGGCCGACACGGTAGCGCCGTGGGTTGCAACCCAGACCTCCATTGGACAAAATTCTTCTACAAATTCTGTGTCTATCCTCAAGTCTTTGATCTGGATTACAAGTGTTTTGATGCAACCCTTCCCTCCTGTGCTTTCCGCATAGTCGAGGAGCATCTCCTCAAGCTAACTGGAGATGAGAGGGTCCCCCGCTACATCGAATCCATACGCCACTCTCGTCACATTTACGGGAGCAACACGTATGAGATGATTGGCGGCAACCCCTCAGGCTGTGTTGGTACTTCCATCATCAACACCATCATCAACAACATTTGTGTCCTTTCTGCTCTGATTCAACATCCAGACTTTTCTCATGACTCCTTCCGGATCCTGGCCTATGGCGATGATGTGATTTACGGATGCGATCCACCCATTCATCCGTCTTACATCAAAGAGTTCTACGACAAGCATACCCCGCTTGTCGTGACTCCCGCCAACAAGGGTTCCACCTTCCCTGACCACTCCACAATCTATGACGTCACGTTTCTGAAACGCTGGTTTGTGCCTGATGATGTCCGTCCATTCTACATCCACCCCGTGATGGACCCAGACACTTACGAGCAGTCAGTGATGTGGCTGAGAGATGGAGACTTCCAGGATCTGGTCACCTCTCTCTGTTTTCTGGCTTTCCACAGTGGCCCGCGTACCTACGCTCGCTGGTGCGACCGCGTCCGCGAGAGGGTCATGCAGACCACTGGCTTTCCACCCACCTTCCTTCCTTACACCTACCTTCAGACTCGCTGGCTGAATTTGCTGGCAGCA

2. Caprine kobuvirus isolate CKoV-China/GCCDC14/2019

ATCATCGTGCCAGGTCCATCCATCGACCCTGGCGTGAATGTCAATCGCAAATCCAAACTCGGTCGCTCCCCCGCTTTTGGTGCCTTTCCAGTCAAGAAACAGCCGGCTCCCCTCACCCAGAAAGACGAACGTCTCGAGCCTGGGATCACCTTGGACGATCAACTCTTCCTTAAACACAACAAGGGAGATATGGATACTCCTTGGCCCGGTCTCGAGGCGGCCGCCGATCTGTACTTTTCCAAATTCCCCACTGTCATTCGCACCCTGACTCTAGAGGAGGCAATCAATGGAACGCCGAACCTCGAAGGCATTGACATGAACCAGGCTGCAGGTTATCCATGGAACACCATGGGTCGCACGCGCCGTTCACTCTTTACTGAAGAGGCACCCGGTTACTACATGCCGACTCCGGAGCTACGGGCTGAGATTGACAAGACCCTTGAAGATCCTCACTACTTCTACTCCACCTTCCTGAAAGACGAGCTCCGTCCGACTTCTAAAGTCGTGGCTGGACTCACCAGAGTGGTCGAGGCAGCTCCCATCCATGCTATCGTTGCTGGCCGCATGTTGCTCGGTGGTCTCATCGAGTACATGCAATCCAATCCTGGCAAGCACGGTAGTGCAGTTGGTTGCAACCCCGACCTGCACTGGACCAAATTCTTCTACAAGTTCTGTCAATACCCCCAAGTCTTTGATCTGGACTACAAGTGTTTTGATGCCACTCTGCCCTCCTGTGCGTTCCGCATAGTTGAGAAGCATCTCCTCAAACTAACTGGAGATGAAAGGGTCCCCCGCTACATCGAATCCATACGCCACTCCCTTCACATCTACGGGAGCAACACGTATGAGATGATTGGCGGAAACCCTTCTGGTTGTGTTGGTACTTCCATCATCAACACCATCATCAACAACATTTGTGTCCTCTCTGCTCTGATCCACCATCCTGACTTCTCTCATGACAGTTACCGGATCCTGGCGTATGGTGATGATGTGATCTATGGGTGCGACCCACCCATCCATCCTTCATACATCAAGGAGTTCTACGACAAGCACACCCCGCTGGTTGTTACTCCCGCCAACAAGGGTTCCACCTTCCCTGAGCACTCCACTATCTATGATGTTACGTTTCTGAAACGCTGGTTTGTGCCCGACGAYGTCCGTCCATTTTACATCCACCCTGTGATGGACCCGGATACCTACGAGCAATCAGTTATGTGGCTGAGAGATGGAGACTTCCAGGATCTGGTTACTTCCCTCTGTTTCCTTGCTTTCCACAGTGGCCCGCGTACCTACGCTCGCTGGTGCGACCGCGTCCGCGAGAGGGTCATGCAGACTACTGGCTTTCCCCCTACTTTCCTTCCCTACACCTACCTCCAAACCCGCTGGCTGAATTTAATGGCTGCA

3. Caprine kobuvirus isolate CKoV-China/SWUN/B12/2022

TCGCTGATTCTCAACCGTGTCAACCTCAAATTCCTGGGCCTTCCTGGTGTCAACATCAATCGGAAGACGCGCCTTAGGCCAAGCCCGGCTTACGGTGCCTTCCCCATCAAGAAGGAACCCGCTCCTCTCAAACGCTCTGATCCCCGCCTCGAGGAGGGCATTGACCTGGACGCCCAGTTGTTTCTTAAACACGATAAGGGCGACCAGTTCAAGCCCTGGCCCTGCTTCGAGGAAGCTTTGGCTCTTTATACCTCCACTCTCCCCTCTTCTTTCCGGACTCTCACACAAGAGGAGGCCATCCATGGAACCCCGTCCATGGAGGGTCTCGACATGAATCAGGCCGCTGGATACCCGTGGAATACTATGGGCCGGTCTCGTCGTTCTCTTTTCGATGAACCGACTCCTGGTGTCTACGTACCAAAACCTGAACTCCAGGTGATGATTGACAGATGCCTGGAGGATCCTGAGTACATCTATTCCACCTTCCTCAAAGATGAACTCAGGCCAATTGACAAGGTGAAGAAGGGCCTGACCCGTGCTGTTGAGTGCGCTCCCATCCACGCTGTCATTGCTGGCCGGATGCTGCTTGGTGGAATCATCGAACACTACCAGGCAAACCCCGGCGAGTATGGCAGCGCGGTTGGCTGCAACCCCGACTACCACTGGACTCCCTTTTTCTACAAATTTGCCAAATATGAAAATGTGTGGGATCTGGATTACAAATGCTTTGATGCTACAATCCCTTCTGTCCTTCTCGGCGCTTACGCCGATTGGGTTGGCCGCGTCACTGGCGACGATCGCGCTCGCCGGTACGTGGAGTCGATCCGCTTTTCTCACCACGTGTACGGGAGTGAACTTTACGACATGCTCGGTGGCAACCCCTCTGGTTGTGTGGGCACGTCTATTATGAACTCCTGGTGCAACAACGTGGCTGTTCTTTCTGCTCTTCTTTATTGCTCTGGCTCTGATTTCAATCCTCGTGATTATGAAATTCTTTGCTACGGCGATGACGTGTTGTACGCGTGTGAGCCTAATGTTCACCCTCGGGACATTAAGGCTTTCTACGACAAGTACACCACGTTTATTGTCACTCCTGCCTCTAAGACTGCTGATTTTCCTGATTCCTCCTCCATCTATGACGTCACGTTCCTGAAGCGCTGGTTCGTCCCGGATGACATTCGCGGGGCTTACATCCATCCCGTGATGCAACCGGACACGTACGAGCAATCAGTGATGTGGATGAAAGATGGAGACTTTCAGTGTATTGTTGATTCTTTGTCTTACTTGGCTTTCCACTCTGGGCCCAAAACCTACGCGGCTTGGTGTCAGAGGGTGGGGGATCAATGTCGGCTCTCCGGAGTCGATGTGCATTTCCTGCCCTATGAATTCCTCCAGGCTAAGTGGGTGAACTTGGTCTCAGCG

4. Caprine kobuvirus isolate CKoV-China/SWUN/F11/2019

ATCATCATTCCGGGACCACCCGTGGATCCCGGGGTGAATGTCAACCGCAAATCCAAACTCGGTCGTTCCCCGGCTTTTGGTGCCTTTCCTGTCAAGAAACAGCCAGCTCCTCTCTCACAGAAAGACGGGCGTCTCGAGGACGGGATCACCTTGGATGACCAACTCTTCCTTAAACACAACAAGGGAGACTTGGATCAGCCATGGCCCGGTCTCGAGGCGGCCGCCGATCTTTACTTCTCCAAATTTCCCACTGTCATTCGAACCCTCACTCTTGAGGAGGCAATCAATGGGACGCCAAACCTGGAAGGCATCGATATGAATCAGGCTGCCGGGTATCCATGGAACACCATGGGACGCTCGCGCCGTTCTCTCTTTGTCGAACAGGCCCCTGGCTTCTACATGCCGACTCCGGAGCTGCAGGAGGAGATTGACAAAACCCTTTCCAACCCAGATTACTTTTACTCCACCTTTCTCAAAGACGAGCTCCGCCCGATCCCCAAAGTGACGGCGGGACTTACCAGAGTGGTCGAGGCTGCTCCCATACATGCTATCGTGGCCGGCCGCATGTTGCTCGGTGGACTCATCGAGTACATGCAGGCACACCCTGGCCGGCACGGTAGCGCCGTGGGTTGCAACCCAGACCTCCATTGGACAAAATTCTTTTATAAATTCTGTGTCTATCCTCAAGTCTTTGATCTGGACTACAAGTGTTTTGATGCAACCCTTCCCTCCTGTGCTTTTCGCATAGTCGAGGAGCATCTCCTCAAACTAACTGGAGATGAAAGGGTCCCCCGCTATATCGAATCCATACGCCACTCCCGTCACATTTACGGGAGCAACACGTATGAGATGATTGGCGGCAACCCTTCAGGCTGTGTTGGTACTTCCATCATCAACACTATCATCAATAACATTTGTGTCCTCTCTGCTCTGATCCAACATCCAGACTTTTCTCATGATTCCTACCGGATCCTGGCCTACGGTGATGATGTGATCTACGGGTGCGATCCGCCCATCCACCCGTCTTACATCAAAGAGTTCTACGATAAGCACACTCCGCTTGTCGTGACTCCCGCCAACAAGGGTTCCACCTTCCCTGAGAGCTCCACAATCTATGACGTCACGTTTCTGAAACGCTGGTTTGTGCCTGATGATGTCCGTCCGTTTTACATCCACCCCGTGATGGACCCAGACACTTACGAGCAGTCGGTGATGTGGCTGAGAGATGGAGACTTCCAGGATCTGGTCACCTCTCTTTGTTTTCTGGCTTTCCACAGTGGCCCGCGTACTTACGCCCGCTGGTGCGACCGCGTCCGCGAGCGGGTCATGCAGACCACTGGTTTTCCTCCTACCTTCCTTCCCTACACCTACCTTCAGACCCGCTGGCTGAATTTGCTGGCAGCG

5. Caprine kobuvirus isolate 12Q108,

ATCATCATCCCGGGCCCCCCTGTGGATCCTGGGGTGAATGTCAACCGCAAATCCAAACTCGGTCGGTCCCCTGCTTTTGGTGCCTTTCCTGTCAAGAAACAGCCAGCCCCTCTTTCTCAGAAAGACGAACGTCTTGAGGACGGGATCACCTTGGATGACCAACTCTTCCTCAAACACAATAAGGGAGATATGGATCAGCCTTGGCCCGGCCTCGAGGCGGCCGCCGATCTCTACTTCTCTAAATTCCCTACCGTCATTCGAACCCTCACTCTAGAGGAGGCGATCAATGGGACGCCAAACCTGGAAGGCATTGACATGAATCAGGCTGCCGGGTATCCATGGAACACCATGGGACGCTCGCGCCGTTCTCTCTTTGTCGAACAGGCCGGCTTCTACATGCCAACTCCGGAGCTGCAGGAGGAGATTGACAAAACTCTTTCCAATCCAGATTACTTTTACTCCACCTTTCTCAAAGACGAGCTCCGCCCGATCCCCAAAGTGACGGCGGGACTCACCAGAGTGGTTGAGGCTGCTCCCATACACGCTATCGTGGCTGGCCGCATGCTGCTCGGTGGTCTCATCGAGTACATGCAGGCACACCCCGGCCGTCATGGTAGCGCTGTGGGCTGCAACCCAGACCTCCATTGGACAAAGTTCTTCTACAAATTCTGTGTCTTTCCTCAAGTCTTTGATTTGGATTACAAGTGTTTTGATGCAACCCTACCCTCCTGTGCCTTTCGCATAGTTGAGGACCATCTCCTCAAACTAACTGGGGATGAGAGGGTTCCCCGCTACATTGAATCCATACGCCACTCTCGCCACATTTACGGGAGCAACACGTATGAGATGATTGGCGGCAACCCTTCAGGTTGTGTTGGTACCTCGATCATCAACACCATCATCAACAACATTTGTGTCCTCTCTGCCCTTATCCAACATCCAGACTTTTCTCATGAATCCTACCGGATCCTGGCCTATGGCGATGATGTGATCTATGGGTGTGATCCACCCATCCACCCGTCTTACATCAAAGAGTTTTACGACAAGCACACTCCGCTCGTCGTGACTCCCGCCAACAAGGGTTCCACCTTCCCTGAGACCTCCACAATCTATGACGTCACGTTTCTGAAACGCTGGTTCGTGCCTGATGATGTCCGTCCATTTTACATCCACCCCGTGATGGACCCAGACACCTACGAGCAGTCAGTCATGTGGCTGAGAGACGGAGACTTCCAGGATCTGGTAACCTCTCTCTGCTTTCTGGCTTTCCACAGTGGCCCGCGTACCTACGCTCGCTGGTGCGATCGCGTCCGCGAGAGGGTCATGCAGACCACTGGCTTTCCACCTACCTTTCTTCCTTACACCTACCTTCAGACCCGCTGGCTGAATCTGCTGGCAGCA

6. Caprine kobuvirus isolate CKoV-China/SWUN/2-JK3/2020 3D

ATCATCATTCCGGGACCACCCGTGGATCCCGGGGTGAATGTCAACCGCAAATCCAAACTCGGTCGTTCCCCGGCTTTTGGTGCCTTTCCTGTCAAGAAACAGCCAGCTCCTCTCTCACAGAAAGACGGGCGTCTCGAGGACGGGATCACCTTGGATGACCAACTCTTCCTTAAACACAACAAGGGAGACTTGGATCAGCCATGGCCCGGTCTCGAGGCGGCCGCCGATCTTTACTTCTCCAAATTTCCCACTGTCATTCGAACCCTCACTCTTGAGGAGGCAATCAATGGGACGCCAAACCTGGAAGGCATCGATATGAATCAGGCTGCCGGGTATCCATGGAACACCATGGGACGCTCGCGCCGTTCTCTCTTTGTCGAACAGGCCCCTGGCTTCTACATGCCGACTCCGGAGCTGCAGGAGGAGATTGACAAAACCCTTTCCAACCCAGATTACTTTTACTCCACCTTTCTCAAAGACGAGCTCCGCCCGATCCCCAAAGTGACGGCGGGACTTACCAGAGTGGTCGAGGCTGCTCCCATACATGCTATCGTGGCCGGCCGCATGTTGCTCGGTGGACTCATCGAGTACATGCAGGCACACCCTGGCCGGCACGGTAGCGCCGTGGGTTGCAACCCAGACCTCCATTGGACAAAATTCTTTTATAAATTCTGTGTCTATCCTCAAGTCTTTGATCTGGACTACAAGTGTTTTGATGCAACCCTTCCCTCCTGTGCTTTTCGCATAGTCGAGGAGCATCTCCTCAAACTAACTGGAGATGAAAGGGTCCCCCGCTATATCGAATCCATACGCCACTCCCGTCACATTTACGGGAGCAACACGTATGAGATGATTGGCGGCAACCCTTCAGGCTGTGTTGGTACTTCCATCATCAACACTATCATCAATAACATTTGTGTCCTCTCTGCTCTGATCCAACATCCAGACTTTTCTCATGATTCCTACCGGATCCTGGCCTACGGTGATGATGTGATCTACGGGTGCGATCCGCCCATCCACCCGTCTTACATCAAAGAGTTCTACGATAAGCACACTCCGCTTGTCGTGACTCCCGCCAACAAGGGTTCCACCTTCCCTGAGAGCTCCACAATCTATGACGTCACGTTTCTGAAACGCTGGTTTGTGCCTGATGATGTCCGTCCGTTTTACATCCACCCCGTGATGGACCCAGACACTTACGAGCAGTCGGTGATGTGGCTGAGAGATGGAGACTTCCAGGATCTGGTCACCTCTCTTTGTTTTCTGGCTTTCCACAGTGGCCCGCGTACTTACGCCCGCTGGTGCGACCGCGTCCGCGAGCGGGTCATGCAGACCACTGGTTTTCCTCCTACCTTCCTTCCCTACACCTACCTTCAGACCCGCTGGCTGAATTTGCTGGCAGCG

7. Caprine kobuvirus isolate CKoV-China/SWUN/1-JK5/2020 3D

ATCATCATTCCGGGACCACCCGTGGATCCCGGGGTGAATGTCAACCGCAAATCCAAACTCGGTCGTTCCCCGGCTTTTGGTGCCTTTCCTGTCAAGAAACAGCCAGCTCCTCTCTCACAGAAAGACGGGCGTCTCGAGGACGGGATCACCTTGGATGACCAACTCTTCCTTAAACACAACAAGGGAGACTTGGATCAGCCATGGCCCGGTCTCGAGGCGGCCGCCGATCTTTACTTCTCCAAATTTCCCACTGTCATTCGAACCCTCACTCTTGAGGAGGCAATCAATGGGACGCCAAACCTGGAAGGCATCGATATGAATCAGGCTGCCGGGTATCCATGGAACACCATGGGACGCTCGCGCCGTTCTCTCTTTGTCGAACAGGCCCCTGGCTTCTACATGCCGACTCCGGAGCTGCAGGAGGAGATTGACAAAACCCTTTCCAACCCAGATTACTTTTACTCCACCTTTCTCAAAGACGAGCTCCGCCCGATCCCCAAAGTGACGGCGGGACTTACCAGAGTGGTCGAGGCTGCTCCCATACATGCTATCGTGGCCGGCCGCATGTTGCTCGGTGGACTCATCGAGTACATGCAGGCACACCCTGGCCGGCACGGTAGCGCCGTGGGTTGCAACCCAGACCTCCATTGGACAAAATTCTTTTATAAATTCTGTGTCTATCCTCAAGTCTTTGATCTGGACTACAAGTGTTTTGATGCAACCCTTCCCTCCTGTGCTTTTCGCATAGTCGAGGAGCATCTCCTCAAACTAACTGGAGATGAAAGGGTCCCCCGCTATATCGAATCCATACGCCACTCCCGTCACATTTACGGGAGCAACACGTATGAGATGATTGGCGGCAACCCTTCAGGCTGTGTTGGTACTTCCATCATCAACACTATCATCAATAACATTTGTGTCCTCTCTGCTCTGATCCAACATCCAGACTTTTCTCATGATTCCTACCGGATCCTGGCCTACGGTGATGATGTGATCTACGGGTGCGATCCGCCCATCCACCCGTCTTACATCAAAGAGTTCTACGATAAGCACACTCCGCTTGTCGTGACTCCCGCCAACAAGGGTTCCACCTTCCCTGAGAGCTCCACAATCTATGACGTCACGTTTCTGAAACGCTGGTTTGTGCCTGATGATGTCCGTCCGTTTTACATCCACCCCGTGATGGACCCAGACACTTACGAGCAGTCGGTGATGTGGCTGAGAGATGGAGACTTCCAGGATCTGGTCACCTCTCTTTGTTTTCTGGCTTTCCACAGTGGCCCGCGTACTTACGCCCGCTGGTGCGACCGCGTCCGCGAGCGGGTCATGCAGACCACTGGTTTTCCTCCTACCTTCCTTCCCTACACCTACCTTCAGACCCGCTGGCTGAATTTGCTGGCAGCG

8. Caprine kobuvirus isolate CKoV-China/SWUN/1-FX2/2020 3D

ATCATCATTCCGGGACCACCCGTGGATCCCGGGGTGAATGTCAACCGCAAATCCAAACTCGGTCGTTCCCCGGCTTTTGGTGCCTTTCCTGTCAAGAAACAGCCAGCTCCTCTCTCACAGAAAGACGGGCGTCTCGAGGACGGGATCACCTTGGATGACCAACTCTTCCTTAAACACAACAAGGGAGACTTGGATCAGCCATGGCCCGGTCTCGAGGCGGCCGCCGATCTTTACTTCTCCAAATTTCCCACTGTCATTCGAACCCTCACTCTTGAGGAGGCAATCAATGGGACGCCAAACCTGGAAGGCATCGATATGAATCAGGCTGCCGGGTATCCATGGAACACCATGGGACGCTCGCGCCGTTCTCTCTTTGTCGAACAGGCCCCTGGCTTCTACATGCCGACTCCGGAGCTGCAGGAGGAGATTGACAAAACCCTTTCCAACCCAGATTACTTTTACTCCACCTTTCTCAAAGACGAGCTCCGCCCGATCCCCAAAGTGACGGCGGGACTTACCAGAGTGGTCGAGGCTGCTCCCATACATGCTATCGTGGCCGGCCGCATGTTGCTCGGTGGACTCATCGAGTACATGCAGGCACACCCTGGCCGGCACGGTAGCGCCGTGGGTTGCAACCCAGACCTCCATTGGACAAAATTCTTTTATAAATTCTGTGTCTATCCTCAAGTCTTTGATCTGGACTACAAGTGTTTTGATGCAACCCTTCCCTCCTGTGCTTTTCGCATAGTCGAGGAGCATCTCCTCAAACTAACTGGAGATGAAAGGGTCCCCCGCTATATCGAATCCATACGCCACTCCCGTCACATTTACGGGAGCAACACGTATGAGATGATTGGCGGCAACCCTTCAGGCTGTGTTGGTACTTCCATCATCAACACTATCATCAATAACATTTGTGTCCTCTCTGCTCTGATCCAACATCCAGACTTTTCTCATGATTCCTACCGGATCCTGGCCTACGGTGATGATGTGATCTACGGGTGCGATCCGCCCATCCACCCGTCTTACATCAAAGAGTTCTACGATAAGCACACTCCGCTTGTCGTGACTCCCGCCAACAAGGGTTCCACCTTCCCTGAGAGCTCCACAATCTATGACGTCACGTTTCTGAAACGCTGGTTTGTGCCTGATGATGTCCGTCCGTTTTACATCCACCCCGTGATGGACCCAGACACTTACGAGCAGTCGGTGATGTGGCTGAGAGATGGAGACTTCCAGGATCTGGTCACCTCTCTTTGTTTTCTGGCTTTCCACAGTGGCCCGCGTACTTACGCCCGCTGGTGCGACCGCGTCCGCGAGCGGGTCATGCAGACCACTGGTTTTCCTCCTACCTTCCTTCCCTACACCTACCTTCAGACCCGCTGGCTGAATTTGCTGGCAGCG

9. Caprine kobuvirus isolate CKoV-China/SWUN/1-FX1/2020 3D

ATCATCATTCCGGGACCACCCGTGGATCCCGGGGTGAATGTCAACCGCAAATCCAAACTCGGTCGTTCCCCGGCTTTTGGTGCCTTTCCTGTCAAGAAACAGCCAGCTCCTCTCTCACAGAAAGACGGGCGTCTCGAGGACGGGATCACCTTGGATGACCAACTCTTCCTTAAACACAACAAGGGAGACTTGGATCAGCCATGGCCCGGTCTCGAGGCGGCCGCCGATCTTTACTTCTCCAAATTTCCCACTGTCATTCGAACCCTCACTCTTGAGGAGGCAATCAATGGGACGCCAAACCTGGAAGGCATCGATATGAATCAGGCTGCCGGGTATCCATGGAACACCATGGGACGCTCGCGCCGTTCTCTCTTTGTCGAACAGGCCCCTGGCTTCTACATGCCGACTCCGGAGCTGCAGGAGGAGATTGACAAAACCCTTTCCAACCCAGATTACTTTTACTCCACCTTTCTCAAAGACGAGCTCCGCCCGATCCCCAAAGTGACGGCGGGACTTACCAGAGTGGTCGAGGCTGCTCCCATACATGCTATCGTGGCCGGCCGCATGTTGCTCGGTGGACTCATCGAGTACATGCAGGCACACCCTGGCCGGCACGGTAGCGCCGTGGGTTGCAACCCAGACCTCCATTGGACAAAATTCTTTTATAAATTCTGTGTCTATCCTCAAGTCTTTGATCTGGACTACAAGTGTTTTGATGCAACCCTTCCCTCCTGTGCTTTTCGCATAGTCGAGGAGCATCTCCTCAAACTAACTGGAGATGAAAGGGTCCCCCGCTATATCGAATCCATACGCCACTCCCGTCACATTTACGGGAGCAACACGTATGAGATGATTGGCGGCAACCCTTCAGGCTGTGTTGGTACTTCCATCATCAACACTATCATCAATAACATTTGTGTCCTCTCTGCTCTGATCCAACATCCAGACTTTTCTCATGATTCCTACCGGATCCTGGCCTACGGTGATGATGTGATCTACGGGTGCGATCCGCCCATCCACCCGTCTTACATCAAAGAGTTCTACGATAAGCACACTCCGCTTGTCGTGACTCCCGCCAACAAGGGTTCCACCTTCCCTGAGAGCTCCACAATCTATGACGTCACGTTTCTGAAACGCTGGTTTGTGCCTGATGATGTCCGTCCGTTTTACATCCACCCCGTGATGGACCCAGACACTTACGAGCAGTCGGTGATGTGGCTGAGAGATGGAGACTTCCAGGATCTGGTCACCTCTCTTTGTTTTCTGGCTTTCCACAGTGGCCCGCGTACTTACGCCCGCTGGTGCGACCGCGTCCGCGAGCGGGTCATGCAGACCACTGGTTTTCCTCCTACCTTCCTTCCCTACACCTACCTTCAGACCCGCTGGCTGAATTTGCTGGCAGCG

10. Caprine kobuvirus isolate CKoV-China/SWUN/HFX3/2020 3D

ATCATCATTCCGGGACCACCCGTGGATCCCGGGGTGAATGTCAACCGCAAATCCAAACTCGGTCGTTCCCCGGCTTTTGGTGCCTTTCCTGTCAAGAAACAGCCAGCTCCTCTTTCACAGAAAGACGGGCGTCTCGAGGACGGGATCACCTTGGATGACCAACTCTTCCTTAAACACAACAAGGGAGACATGGATCAGCCATGGCCCGGTCTCGAGGCGGCCGCCGATCTTTACTTCTCCAAATTTCCCACTGTCATTCGAACCCTCACTCTTGAGGAGGCAATCAATGGGACGCCAAACCTGGAAGGCATCGATATGAATCAGGCTGCCGGGTATCCATGGAACACCATGGGACGCTCGCGCCGTTCTCTCTTTGTCGAACAGGCCCCTGGCTTCTACATGCCGACTCCGGAGCTACAGGAGGAGATTGATAAAACCCTTTCCAACCCAGATTACTTTTACTCCACCTTTCTCAAAGACGAGCTCCGCCCGATCCCCAAAGTGACGGCGGGACTTACCAGAGTGGTCGAGGCTGCTCCCATACATGCTATCGTGGCCGGCCGCATGTTGCTCGGTGGACTCATCGAGTACATGCAGGCACACCCTGGCCGGCACGGTAGCGCCGTGGGTTGCAACCCAGACCTCCATTGGACAAAATTCTTTTATAAATTCTGTGTCTATCCTCAAGTCTTTGATCTGGACTACAAGTGTTTTGATGCAACCCTTCCCTCCTGTGCTTTTCGCATAGTCGAGGAGCATCTCCTCAAACTAACTGGAGATGAAAGGGTCCCCCGCTATATCGAATCCATACGCCACTCCCGTCACATTTACGGGAGCAACACGTATGAGATGATTGGCGGCAACCCTTCAGGCTGTGTTGGTACTTCCATCATCAACACTATCATCAATAACATTTGTGTCCTCTCTGCTCTGATCCAACATCCAGACTTTTCTCATGATTCCTACCGGATCCTGGCCTACGGTGATGATGTGATCTACGGGTGCGATCCGCCCATCCACCCGTCTTACATCAAAGAGTTCTACGATAAGCACACTCCGCTTGTCGTGACTCCCGCCAACAAGGGTTCCACCTTCCCTGAGAGCTCCACAATCTATGACGTCACGTTTCTGAAACGCTGGTTTGTGCCTGATGATGTCCGTCCGTTTTACATCCACCCCGTGATGGACCCAGACACTTACGAGCAGTCGGTGATGTGGCTGAGAGATGGAGACTTCCAGGATCTGGTCACCTCTCTTTGTTTTCTGGCTTTCCACAGTGGCCCGCGTACTTACGCCCGCTGGTGCGACCGCGTCCGCGAGCGGGTCATGCAGACCACTGGTTTTCCTCCTACCTTCCTTCCCTACACCTACCTTCAGACCCGCTGGCTGAATTTGCTGGCAGCG

11. Caprine kobuvirus isolate CKoV-China/SWUN/HFX6/2020 3D

ATCATCATTCCGGGACCACCCGTGGATCCCGGGGTGAATGTCAACCGCAAATCCAAACTCGGTCGTTCCCCGGCTTTTGGTGCCTTTCCTGTCAAGAAACAGCCAGCTCCTCTTTCACAGAAAGACGGGCGTCTCGAGGACGGGATCACCTTGGATGACCAACTCTTCCTTAAACACAACAAGGGAGACATGGATCAGCCATGGCCCGGTCTCGAGGCGGCCGCCGATCTTTACTTCTCCAAATTTCCCACTGTCATTCGAACCCTCACTCTTGAGGAGGCAATCAATGGGACGCCAAACCTGGAAGGCATCGATATGAATCAGGCTGCCGGGTATCCATGGAACACCATGGGACGCTCGCGCCGTTCTCTCTTTGTCGAACAGGCCCCTGGCTTCTACATGCCGACTCCGGAGCTACAGGAGGAGATTGATAAAACCCTTTCCAACCCAGATTACTTTTACTCCACCTTTCTCAAAGACGAGCTCCGCCCGATCCCCAAAGTGACGGCGGGACTTACCAGAGTGGTCGAGGCTGCTCCCATACATGCTATCGTGGCCGGCCGCATGTTGCTCGGTGGACTCATCGAGTACATGCAGGCACACCCTGGCCGGCACGGTAGCGCCGTGGGTTGCAACCCAGACCTCCATTGGACAAAATTCTTTTATAAATTCTGTGTCTATCCTCAAGTCTTTGATCTGGACTACAAGTGTTTTGATGCAACCCTTCCCTCCTGTGCTTTTCGCATAGTCGAGGAGCATCTCCTCAAACTAACTGGAGATGAAAGGGTCCCCCGCTATATCGAATCCATACGCCACTCCCGTCACATTTACGGGAGCAACACGTATGAGATGATTGGCGGCAACCCTTCAGGCTGTGTTGGTACTTCCATCATCAACACTATCATCAATAACATTTGTGTCCTCTCTGCTCTGATCCAACATCCAGACTTTTCTCATGATTCCTACCGGATCCTGGCCTACGGTGATGATGTGATCTACGGGTGCGATCCGCCCATCCACCCGTCTTACATCAAAGAGTTCTACGATAAGCACACTCCGCTTGTCGTGACTCCCGCCAACAAGGGTTCCACCTTCCCTGAGAGCTCCACAATCTATGACGTCACGTTTCTGAAACGCTGGTTTGTGCCTGATGATGTCCGTCCGTTTTACATCCACCCCGTGATGGACCCAGACACTTACGAGCAGTCGGTGATGTGGCTGAGAGATGGAGACTTCCAGGATCTGGTCACCTCTCTTTGTTTTCTGGCTTTCCACAGTGGCCCGCGTACTTACGCCCGCTGGTGCGACCGCGTCCGCGAGCGGGTCATGCAGACCACTGGTTTTCCTCCTACCTTCCTTCCCTACACCTACCTTCAGACCCGCTGGCTGAATTTGCTGGCAGCG

12. Caprine kobuvirus isolate CKoV-China/SWUN/HJK7/2020 3D

ATCATCATTCCGGGACCACCCGTGGATCCCGGGGTGAATGTCAACCGCAAATCCAAACTCGGTCGTTCCCCGGCTTTTGGTGCCTTTCCTGTCAAGAAACAGCCAGCTCCTCTTTCACAGAAAGACGGGCGTCTCGAGGACGGGATCACCTTGGATGACCAACTCTTCCTTAAACACAACAAGGGAGACATGGATCAGCCATGGCCCGGTCTCGAGGCGGCCGCCGATCTTTACTTCTCCAAATTTCCCACTGTCATTCGAACCCTCACTCTTGAGGAGGCAATCAATGGGACGCCAAACCTGGAAGGCATCGATATGAATCAGGCTGCCGGGTATCCATGGAACACCATGGGACGCTCGCGCCGTTCTCTCTTTGTCGAACAGGCCCCTGGCTTCTACATGCCGACTCCGGAGCTACAGGAGGAGATTGATAAAACCCTTTCCAACCCAGATTACTTTTACTCCACCTTTCTCAAAGACGAGCTCCGCCCGATCCCCAAAGTGACGGCGGGACTTACCAGAGTGGTCGAGGCTGCTCCCATACATGCTATCGTGGCCGGCCGCATGTTGCTCGGTGGACTCATCGAGTACATGCAGGCACACCCTGGCCGGCACGGTAGCGCCGTGGGTTGCAACCCAGACCTCCATTGGACAAAATTCTTTTATAAATTCTGTGTCTATCCTCAAGTCTTTGATCTGGACTACAAGTGTTTTGATGCAACCCTTCCCTCCTGTGCTTTTCGCATAGTCGAGGAGCATCTCCTCAAACTAACTGGAGATGAAAGGGTCCCCCGCTATATCGAATCCATACGCCACTCCCGTCACATTTACGGGAGCAACACGTATGAGATGATTGGCGGCAACCCTTCAGGCTGTGTTGGTACTTCCATCATCAACACTATCATCAATAACATTTGTGTCCTCTCTGCTCTGATCCAACATCCAGACTTTTCTCATGATTCCTACCGGATCCTGGCCTACGGTGATGATGTGATCTACGGGTGCGATCCGCCCATCCACCCGTCTTACATCAAAGAGTTCTACGATAAGCACACTCCGCTTGTCGTGACTCCCGCCAACAAGGGTTCCACCTTCCCTGAGAGCTCCACAATCTATGACGTCACGTTTCTGAAACGCTGGTTTGTGCCTGATGATGTCCGTCCGTTTTACATCCACCCCGTGATGGACCCAGACACTTACGAGCAGTCGGTGATGTGGCTGAGAGATGGAGACTTCCAGGATCTGGTCACCTCTCTTTGTTTTCTGGCTTTCCACAGTGGCCCGCGTACTTACGCCCGCTGGTGCGACCGCGTCCGCGAGCGGGTCATGCAGACCACTGGTTTTCCTCCTACCTTCCTTCCCTACACCTACCTTCAGACCCGCTGGCTGAATTTGCTGGCAGCG

13. Caprine kobuvirus isolate CKoV-China/SWUN/F6/2020 3D

ATCATCATTCCGGGACCACCCGTGGATCCCGGGGTGAATGTCAACCGCAAATCCAAACTCGGTCGTTCCCCGGCTTTTGGTGCCTTTCCTGTCAAGAAACAGCCAGCTCCTCTTTCACAGAAAGACGGGCGTCTCGAGGACGGGATCACCTTGGATGACCAACTCTTCCTTAAACACAACAAGGGAGACATGGATCAGCCATGGCCCGGTCTCGAGGCGGCCGCCGATCTTTACTTCTCCAAATTTCCCACTGTCATTCGAACCCTCACTCTTGAGGAGGCAATCAATGGGACGCCAAACCTGGAAGGCATCGATATGAATCAGGCTGCCGGGTATCCATGGAACACCATGGGACGCTCGCGCCGTTCTCTCTTTGTCGAACAGGCCCCTGGCTTCTACATGCCGACTCCGGAGCTACAGGAGGAGATTGACAAAACCCTTTCCAACCCAGATTACTTTTATTCCACCTTTCTCAAAGACGAGCTCCGCCCGATCTCCAAAGTGACGGCGGGACTTACCAGAGTGGTCGAGGCTGCTCCCATACATGCTATCGTGGCCGGCCGCATGTTGCTCGGTGGACTCATCGAGTACATGCAGGCACACCCTGGCCGGCACGGTAGCGCCGTGGGTTGCAACCCAGACCTCCATTGGACAAAATTCTTTTATAAATTCTGTGTCTATCCTCAAGTCTTTGATCTGGACTACAAGTGTTTTGATGCAACCCTTCCCTCCTGTGCTTTTCGCATAGTCGAGGAGCATCTCCTCAAACTAACTGGAGATGAAAGGGTCCCCCGCTATATCGAATCCATACGCCACTCCCGTCACATTTACGGGAGCAACACGTATGAGATGATTGGCGGCAACCCTTCAGGCTGTGTTGGTACTTCCATCATCAACACTATCATCAATAACATTTGTGTCCTCTCTGCTCTGATCCAACATCCAGACTTTTCTCATGATTCCTACCGGATCCTGGCCTACGGTGATGATGTGATCTACGGGTGCGATCCGCCCATCCACCCGTCTTACATCAAAGAGTTCTACGATAAGCACACTCCGCTTGTCGTGACTCCCGCCAACAAGGGTTCCACCTTCCCTGAGAGCTCCACAATCTATGACGTCACGTTTCTGAAACGCTGGTTTGTGCCTGATGATGTCCGTCCGTTTTACATCCACCCCGTGATGGACCCAGACACTTACGAGCAGTCGGTGATGTGGCTGAGAGATGGAGACTTCCAGGATCTGGTCACCTCTCTTTGTTTTCTGGCTTTCCACAGTGGCCCGCGTACTTACGCCCGCTGGTGCGACCGCGTCCGCGAGCGGGTCATGCAGACCACTGGTTTTCCTCCTACCTTCCTTCCCTACACCTACCTTCAGACCCGCTGGCTGAATTTGCTGGCAGCG

14. Caprine kobuvirus isolate CKoV-China/SWUN/24/2020 3D

ATCATCATTCCGGGACCACCCGTGGATCCCGGGGTGAATGTCAACCGCAAATCCAAACTCGGTCGTTCCCCGGCTTTTGGTGCCTTTCCTGTCAAGAAACAGCCAGCTCCTCTTTCACAGAAAGACGGGCGTCTCGAGGACGGGATCACCTTGGATGACCAACTCTTCCTTAAACACAACAAGGGAGACATGGATCAGCCATGGCCCGGTCTCGAGGCGGCCGCCGATCTTTACTTCTCCAAATTTCCCACTGTCATTCGAACCCTCACTCTTGAGGAGGCAATCAATGGGACGCCAAACCTGGAAGGCATCGATATGAATCAGGCTGCCGGGTATCCATGGAACACCATGGGACGCTCGCGCCGTTCTCTCTTTGTCGAACAGGCCCCTGGCTTCTACATGCCGACTCCGGAGCTGCAGGAGGAGATTGACAAAACCCTTTCCAACCCAGATTACTTTTACTCCACCTTTCTCAAAGACGAGCTCCGCCCGATCCCCAAAGTGACGGCGGGACTTACCAGAGTGGTCGAGGCTGCTCCCATACATGCTATCGTGGCCGGCCGCATGTTGCTCGGTGGACTCATCGAGTACATGCAGGCACACCCTGGCCGGCACGGTAGCGCCGTGGGTTGCAACCCAGACCTCCATTGGACAAAATTCTTTTATAAATTCTGTGTCTATCCTCAAGTCTTTGATCTGGACTACAAGTGTTTTGATGCAACCCTTCCCTCCTGTGCTTTTCGCATAGTCGAGGAGCATCTCCTCAAACTAACTGGAGATGAAAGGGTCCCCCGCTATATCGAATCCATACGCCACTCCCGTCACATTTACGGGAGCAACACGTATGAGATGATTGGCGGCAACCCTTCAGGCTGTGTTGGTACTTCCATCATCAACACTATCATCAATAACATTTGTGTCCTCTCTGCTCTGATCCAACATCCAGACTTTTCTCATGATTCCTACCGGATCCTGGCCTACGGTGATGATGTGATCTACGGGTGCGATCCGCCCATCCACCCGTCTTACATCAAAGAGTTCTACGATAAGCACACTCCGCTTGTCGTGACTCCCGCCAACAAGGGTTCCACCTTCCCTGAGAGCTCCACAATCTATGACGTCACGTTTCTGAAACGCTGGTTTGTGCCTGATGATGTCCGTCCGTTTTACATCCACCCCGTGATGGACCCAGACACTTACGAGCAGTCGGTGATGTGGCTGAGAGATGGAGACTTCCAGGATCTGGTCACCTCTCTTTGTTTTCTGGCTTTCCACAGTGGCCCGCGTACTTACGCCCGCTGGTGCGACCGCGTCCGCGAGCGGGTCATGCAGACCACTGGTTTTCCTCCTACCTTCCTTCCCTACACCTACCTTCAGACCCGCTGGCTGAATTTGCTGGCAGCG

15. Caprine kobuvirus isolate CKoV-China/SWUN/25/2020 3D

ATCATCATTCCGGGACCACCCGTGGATCCCGGGGTGAATGTCAACCGCAAATCCAAACTCGGTCGTTCCCCGGCTTTTGGTGCCTTTCCTGTCAAGAAACAGCCAGCTCCTCTCTCACAGAAAGACGGGCGTCTCGAGGACGGGATCACCTTGGATGACCAACTCTTCCTTAAACACAACAAGGGAGACTTGGATCAGCCATGGCCCGGTCTCGAGGCGGCCGCCGATCTTTACTTCTCCAAATTTCCCACTGTCATTCGAACCCTCACTCTTGAGGAGGCAATCAATGGGACGCCAAACCTGGAAGGCATCGATATGAATCAGGCTGCCGGGTATCCATGGAACACCATGGGACGCTCGCGCCGTTCTCTCTTTGTCGAACAGGCCCCTGGCTTCTACATGCCGACTCCGGAGCTGCAGGAGGAGATTGACAAAACCCTTTCCAACCCAGATTACTTTTACTCCACCTTTCTCAAAGACGAGCTCCGCCCGATCCCCAAAGTGACGGCGGGACTTACCAGAGTGGTCGAGGCTGCTCCCATACATGCTATCGTGGCCGGCCGCATGTTGCTCGGTGGACTCATCGAGTACATGCAGGCACACCCTGGCCGGCACGGTAGCGCCGTGGGTTGCAACCCAGACCTCCATTGGACAAAATTCTTTTATAAATTCTGTGTCTATCCTCAAGTCTTTGATCTGGACTACAAGTGTTTTGATGCAACCCTTCCCTCCTGTGCTTTTCGCATAGTCGAGGAGCATCTCCTCAAACTAACTGGAGATGAAAGGGTCCCCCGCTATATCGAATCCATACGCCACTCCCGTCACATTTACGGGAGCAACACGTATGAGATGATTGGCGGCAACCCTTCAGGCTGTGTTGGTACTTCCATCATCAACACTATCATCAATAACATTTGTGTCCTCTCTGCTCTGATCCAACATCCAGACTTTTCTCATGATTCCTACCGGATCCTGGCCTACGGTGATGATGTGATCTACGGGTGCGATCCGCCCATCCACCCGTCTTACATCAAAGAGTTCTACGATAAGCACACTCCGCTTGTCGTGACTCCCGCCAACAAGGGTTCCACCTTCCCTGAGAGCTCCACAATCTATGACGTCACGTTTCTGAAACGCTGGTTTGTGCCTGATGATGTCCGTCCGTTTTACATCCACCCCGTGATGGACCCAGACACTTACGAGCAGTCGGTGATGTGGCTGAGAGATGGAGACTTCCAGGATCTGGTCACCTCTCTTTGTTTTCTGGCTTTCCACAGTGGCCCGCGTACTTACGCCCGCTGGTGCGACCGCGTCCGCGAGCGGGTCATGCAGACCACTGGTTTTCCTCCTACCTTCCTTCCCTACACCTACCTTCAGACCCGCTGGCTGAATTTGCTGGCAGCG

16. Caprine kobuvirus isolate CKoV-China/SWUN/F5/2020 3D

ATCATCATTCCGGGACCACCCGTGGATCCCGGGGTGAATGTCAACCGCAAATCCAAACTCGGTCGTTCCCCGGCTTTTGGTGCCTTTCCTGTCAAGAAACAGCCAGCTCCTCTTTCACAGAAAGACGGGCGTCTCGAGGACGGGATCACCTTGGATGACCAACTCTTCCTTAAACACAACAAGGGAGACATGGATCAGCCATGGCCCGGTCTCGAGGCGGCCGCCGATCTTTACTTCTCCAAATTTCCCACTGTCATTCGAACCCTCACTCTTGAGGAGGCAATCAATGGGACGCCAAACCTGGAAGGCATCGATATGAATCAGGCTGCCGGGTATCCATGGAACACCATGGGACGCTCGCGCCGTTCTCTCTTTGTCGAACAGGCCCCTGGCTTCTACATGCCGACTCCGGAGCTGCAGGAGGAGATTGACAAAACCCTTTCCAACCCAGATTACTTTTACTCCACCTTTCTCAAAGACGAGCTCCGCCCGATCCCCAAAGTGACGGCGGGACTTACCAGAGTGGTCGAGGCTGCTCCCATACATGCTATCGTGGCCGGCCGCATGTTGCTCGGTGGACTCATCGAGTACATGCAGGCACACCCTGGCCGGCACGGTAGCGCCGTGGGTTGCAACCCAGACCTCCATTGGACAAAATTCTTTTATAAATTCTGTGTCTATCCTCAAGTCTTTGATCTGGACTACAAGTGTTTTGATGCAACCCTTCCCTCCTGTGCTTTTCGCATAGTCGAGGAGCATCTCCTCAAACTAACTGGAGATGAAAGGGTCCCCCGCTATATCGAATCCATACGCCACTCCCGTCACATTTACGGGAGCAACACGTATGAGATGATTGGCGGCAACCCTTCAGGCTGTGTTGGTACTTCCATCATCAACACTATCATCAATAACATTTGTGTCCTCTCTGCTCTGATCCAACATCCAGACTTTTCTCATGATTCCTACCGGATCCTGGCCTACGGTGATGATGTGATCTACGGGTGCGATCCGCCCATCCACCCGTCTTACATCAAAGAGTTCTACGATAAGCACACTCCGCTTGTCGTGACTCCCGCCAACAAGGGTTCCACCTTCCCTGAGAGCTCCACAATCTATGACGTCACGTTTCTGAAACGCTGGTTTGTGCCTGATGATGTCCGTCCGTTTTACATCCACCCCGTGATGGACCCAGACACTTACGAGCAGTCGGTGATGTGGCTGAGAGATGGAGACTTCCAGGATCTGGTCACCTCTCTTTGTTTTCTGGCTTTCCACAGTGGCCCGCGTACTTACGCCCGCTGGTGCGACCGCGTCCGCGAGCGGGTCATGCAGACCACTGGTTTTCCTCCTACCTTCCTTCCCTACACCTACCTTCAGACCCGCTGGCTGAATTTGCTGGCAGCG

17. Caprine kobuvirus isolate CKoV-China/SWUN/F8/2020 3D

ATCATCATTCCGGGACCACCCGTGGATCCCGGGGTGAATGTCAACCGCAAATCCAAACTCGGTCGTTCCCCGGCTTTTGGTGCCTTTCCTGTCAAGAAACAGCCAGCTCCTCTTTCACAGAAAGACGGGCGTCTCGAGGACGGGATCACCTTGGATGACCAACTCTTCCTTAAACACAACAAGGGAGACATGGATCAGCCATGGCCCGGTCTCGAGGCGGCCGCCGATCTTTACTTCTCCAAATTTCCCACTGTCATTCGAACCCTCACTCTTGAGGAGGCAATCAATGGGACGCCAAACCTGGAAGGCATCGATATGAATCAGGCTGCCGGGTATCCATGGAACACCATGGGACGCTCGCGCCGTTCTCTCTTTGTCGAACAGGCCCCTGGCTTCTACATGCCGACTCCGGAGCTGCAGGAGGAGATTGACAAAACCCTTTCCAACCCAGATTACTTTTACTCCACCTTTCTCAAAGACGAGCTCCGCCCGATCCCCAAAGTGACGGCGGGACTTACCAGAGTGGTCGAGGCTGCTCCCATACATGCTATCGTGGCCGGCCGCATGTTGCTCGGTGGACTCATCGAGTACATGCAGGCACACCCTGGCCGGCACGGTAGCGCCGTGGGTTGCAACCCAGACCTCCATTGGACAAAATTCTTTTATAAATTCTGTGTCTATCCTCAAGTCTTTGATCTGGACTACAAGTGTTTTGATGCAACCCTTCCCTCCTGTGCTTTTCGCATAGTCGAGGAGCATCTCCTCAAACTAACTGGAGATGAAAGGGTCCCCCGCTATATCGAATCCATACGCCACTCCCGTCACATTTACGGGAGCAACACGTATGAGATGATTGGCGGCAACCCTTCAGGCTGTGTTGGTACTTCCATCATCAACACTATCATCAATAACATTTGTGTCCTCTCTGCTCTGATCCAACATCCAGACTTTTCTCATGATTCCTACCGGATCCTGGCCTACGGTGATGATGTGATCTACGGGTGCGATCCGCCCATCCACCCGTCTTACATCAAAGAGTTCTACGATAAGCACACTCCGCTTGTCGTGACTCCCGCCAACAAGGGTTCCACCTTCCCTGAGAGCTCCACAATCTATGACGTCACGTTTCTGAAACGCTGGTTTGTGCCTGATGATGTCCGTCCGTTTTACATCCACCCCGTGATGGACCCAGACACTTACGAGCAGTCGGTGATGTGGCTGAGAGATGGAGACTTCCAGGATCTGGTCACCTCTCTTTGTTTTCTGGCTTTCCACAGTGGCCCGCGTACTTACGCCCGCTGGTGCGACCGCGTCCGCGAGCGGGTCATGCAGACCACTGGTTTTCCTCCTACCTTCCTTCCCTACACCTACCTTCAGACCCGCTGGCTGAATTTGCTGGCAGCG

18. Caprine kobuvirus isolate CKoV-China/SWUN/F7/2020 3D

ATCATCATTCCGGGACCACCCGTGGATCCCGGGGTGAATGTCAACCGCAAATCCAAACTCGGTCGTTCCCCGGCTTTTGGTGCCTTTCCTGTCAAGAAACAGCCAGCTCCTCTTTCACAGAAAGACGGGCGTCTCGAGGACGGGATCACCTTGGATGACCAACTCTTCCTTAAACACAACAAGGGAGACATGGATCAGCCATGGCCCGGTCTCGAGGCGGCCGCCGATCTTTACTTCTCCAAATTTCCCACTGTCATTCGAACCCTCACTCTTGAGGAGGCAATCAATGGGACGCCAAACCTGGAAGGCATCGATATGAATCAGGCTGCCGGGTATCCATGGAACACCATGGGACGCTCGCGCCGTTCTCTCTTTGTCGAACAGGCCCCTGGCTTCTACATGCCGACTCCGGAGCTGCAGGAGGAGATTGACAAAACCCTTTCCAACCCAGATTACTTTTACTCCACCTTTCTCAAAGACGAGCTCCGCCCGATCCCCAAAGTGACGGCGGGACTTACCAGAGTGGTCGAGGCTGCTCCCATACATGCTATCGTGGCCGGCCGCATGTTGCTCGGTGGACTCATCGAGTACATGCAGGCACACCCTGGCCGGCACGGTAGCGCCGTGGGTTGCAACCCAGACCTCCATTGGACAAAATTCTTTTATAAATTCTGTGTCTATCCTCAAGTCTTTGATCTGGACTACAAGTGTTTTGATGCAACCCTTCCCTCCTGTGCTTTTCGCATAGTCGAGGAGCATCTCCTCAAACTAACTGGAGATGAAAGGGTCCCCCGCTATATCGAATCCATACGCCACTCCCGTCACATTTACGGGAGCAACACGTATGAGATGATTGGCGGCAACCCTTCAGGCTGTGTTGGTACTTCCATCATCAACACTATCATCAATAACATTTGTGTCCTCTCTGCTCTGATCCAACATCCAGACTTTTCTCATGATTCCTACCGGATCCTGGCCTACGGTGATGATGTGATCTACGGGTGCGATCCGCCCATCCACCCGTCTTACATCAAAGAGTTCTACGATAAGCACACTCCGCTTGTCGTGACTCCCGCCAACAAGGGTTCCACCTTCCCTGAGAGCTCCACAATCTATGACGTCACGTTTCTGAAACGCTGGTTTGTGCCTGATGATGTCCGTCCGTTTTACATCCACCCCGTGATGGACCCAGACACTTACGAGCAGTCGGTGATGTGGCTGAGAGATGGAGACTTCCAGGATCTGGTCACCTCTCTTTGTTTTCTGGCTTTCCACAGTGGCCCGCGTACTTACGCCCGCTGGTGCGACCGCGTCCGCGAGCGGGTCATGCAGACCACTGGTTTTCCTCCTACCTTCCTTCCCTACACCTACCTTCAGACCCGCTGGCTGAATTTGCTGGCAGCG

19. Caprine kobuvirus strain OBI/2017/TUR 3D

TGGATTACAAGTGTTTTGATGCAACCCTGCCCTCCTGTGCCTTCCGCATAGTCGAGGACCATCTCCTCAAGCTAACTGGAGATGAGAGGGTTCCCCGCTACATCGAATCCATACGCCACTCCCGCCACATCTACGGGAGTAACACGTATGAGATGATTGGCGGCAACCCTTCGGGTTGTGTTGGTACTTCCATCATCAACACCATCATCAACAACAT

20. Caprine kobuvirus isolate MN/2019 3D

AACACAACAAGGGAGATATGGATCAGCCTTGGCCCGGTCTCGAGGCAGCCGCCGATCTCTACTTTTCTAAATTTCCTACCGTCATTCGAACCCTCACTCTAGAGGAGGCGATCAATGGGACGCCAAACCTGGAAGGCATTGATATGAATCAGGCTGCCGGGTATCCATGGAATACCATGGGACGCTCGCGCCGTTCTCTCTTTGTCGAACAGGCCCCTGGCTTCTACATGCCAACTCCGGAGCTGCAGGAAGAGATTGACAAGACCCTTTCCAATCCAGATTACTTCTATTCCACCTTTCTCAAAGACGAGCTCCGCCCGATCTCCAAAGTGACGGCGGGACTCACCAGAGTGGTCGAAGCTGCTCCCATACACGCTATCGTGGCTGGCCGCATGTTGCTCGGTGGTCTCATCGAGTACATGCAAGCACATCCCGGTCGTCATGGTAGCGCCGTGGGCTGCAACCCAGACCTCCATTGGACAAAGTTTTTCTACAAATTCTGTGTCTACCCTCAAGTCTTTGATCTGGACTACAAGTGTTTTGATGCAACTCTGCCCTCCTGTGCTTTCCGCATAGTTGAGGACCATCTCCTCAAGCTAACTGGAGATGAGAGGGTTCCCCGCTACATCGAATCCATACGCCACTCCCGCCACATTTACGGGAGCAACACGTATGAG

21. Caprine kobuvirus isolate 51R/IT 3D

TCCTCAAGTCATAGGTTGGGATTTCAAGTGTTTTGATGCAACCCTCCCCTCCTGTGCTTTTCGCATAGTTGAGGAAGATCTCCTCAAACTAACTGGAGATGAACGGGTGCCCCGCTATATCGAGTCCATACGACACTCCCGTCACATTTACGGGAGTTCGACGTATGAGATGATTGGTGGGAATCCCTCAGGGTGTGTTGGTACTTCCATCATCAACACCATCAT

22. Caprine kobuvirus isolate 61R/IT 3D

GATTACAAGTGTTTTGATGCCACCCTCCCCTCCTGTGCCTTTCGCATAGTTGAGGACCATCTCCTCAAGATCACTGGAGATGAACGGGTGCCCCGCTACATTGAGTCCATACGACACTCCCGTCATGTTTACGGGAGTTCGACGTATGAGATGATTGGCGGAAACCCCTCAGGGTGTGTTGGTACTTCCATCATCAACACCATCAA

23. Caprine kobuvirus isolate 71R/IT 3D

GGATTACAAGTGTTTTGATGCCACCCTCCCCTCCTGTGCCTTTCGCATAGTTGAGGAACATCTCCTCAAGATCATAGGACATGAACGGGTGCCCCGCTATATCGAGTCCATAACTAACTCCCGTCATATCTACGGGAGCTCGACGTATGAGATGATCGGCGGAAACCCCTCAGGGTGTGTTGGTACTTCCATCATCAACACCATCA

24. Caprine kobuvirus isolate 50R/IT 3D

GGATTACAAGTGTTTTGATGCAACCCTCCCCTCCTGTGCTTTTCGCATAGTTGAGGACCATCTCCTTAAGATTATAGGAGATGAACGGGTGCCCCGCTACATTGAATCCATACGACACTCCCGTCACATCTACGGGAGTTCGACGTATGAGATGATTGGTGGGAATCCCTCAGGGTGTGTTGGTACTTCCATCATCAACACCATCA

Figure S1. Optimization of primer concentration. The images show the results of iiPCR amplification using different concentrations of forward and reverse primers (10 μmol/μL) at volumes ranging from 0.5 μL to 4 μL. Tubes 1–8 correspond to primer volumes of 0.5 μL, 1.0 μL, 1.5 μL, 2.0 μL, 2.5 μL, 3.0 μL, 3.5 μL, and 4.0 μL, respectively. Positive results are indicated by a "+" sign, and negative results are indicated by a "-" sign.

Figure S2. Optimization of probe concentration. The images show the results of iiPCR amplification using different concentrations of the probe (10 μmol/μL) at volumes ranging from 0.5 μL to 2.5 μL. Tubes 1–5 correspond to probe volumes of 0.5 μL, 1.0 μL, 1.5 μL, 2.0 μL, and 2.5 μL, respectively. Tubes 6–8 represent negative controls. Positive results are indicated by a "+" sign, and negative results are indicated by a "-" sign.

Figure S3. Optimization of template concentration. The images show the results of iiPCR amplification using different template volumes ranging from 0.5 μL to 4 μL. Tubes 1–8 correspond to template volumes of 0.5 μL, 1.0 μL, 1.5 μL, 2.0 μL, 2.5 μL, 3.0 μL, 3.5 μL, and 4.0 μL, respectively. Positive results are indicated by a "+" sign, and negative results are indicated by a "-" sign.
